# Supplementary material for: Attribute identification based IoT fog data security control and forwarding
Source: PeerJ Comput Sci. 2023 Dec 20;9:e1747. doi: 10.7717/peerj-cs.1747 (PMC10773925; doi:10.7717/peerj-cs.1747)
Supplement: Supplemental Information 1 [file peerj-cs-09-1747-s001.docx]

The experimental data required in the paper is generated by scapy and stored in PCAP format. *experiment*1*.pcap* through *experiment*6*.pcap* each store the data packets corresponding to Experiments 1 through 6, respectively.And *pcap* files can be read using the rdpcap() function in the scapy library.

*experiment*1*.pcap* contains 5000 packets, of which 1000 are each FlowA to FlowE.

*experiment*2*.pcap* contains 10000 packets, of which 7000 are security packets, 1000 are illegally accessed packets, 1000 are malicious tampering packets and 1000 are forged matching packets.

*experiment*3*.pcap* contains 5000 packets, with 500 packets each with the feature number of 5, 10, 15, 20, 25, and 30.

*experiment*4*.pcap* contains 2000 packets, including 1000 regular packets and 1000 packets containing legal attribute identification.

*experiment*5*.pcap* contains 2000 packets, including 1000 regular packets and 1000 packets containing legal attribute identification.

*experiment*6*.pcap* contains 3000 packets, including 1000 regular packets and 2000 packets containing legal attribute identification.
